# Mitochondria–immunometabolism in sepsis-induced liver injury: mechanisms, biomarkers, and therapeutic opportunities

**DOI:** 10.3389/fcimb.2026.1866390

**Published:** 2026-09-02

**Authors:** Yi Wang, Pingsen Zhao

**Affiliations:** 1 Department of Laboratory Medicine, Yuebei People’s Hospital, Shantou University Medical College, Shaoguan, China; 2 Laboratory for Diagnosis of Clinical Microbiology and Infection, Yuebei People’s Hospital, Shantou University Medical College, Shaoguan, China; 3 Research Center for Interdisciplinary & High-quality Innovative Development in Laboratory Medicine, Shaoguan, China; 4 Shaoguan Municipal Quality Control Center for Laboratory Medicine, Yuebei People’s Hospital Affiliated to Shantou University Medical College, Yuebei People’s Hospital Affiliated to Shantou University Medical College, Shaoguan, China; 5 Shaoguan Municipal Quality Control Center for Surveillance of Bacterial Resistance, Yuebei People’s Hospital Affiliated to Shantou University Medical College, Shaoguan, China; 6 Shaoguan Engineering Research Center for Research and Development of Molecular and Cellular Technology in Rapid Diagnosis of Infectious Diseases and Cancer, Yuebei People’s Hospital Affiliated to Shantou University Medical College, Shaoguan, China

**Keywords:** biomarkers, mechanisms, sepsis, sepsis-induced liver injury, therapeutics

## Abstract

Sepsis-induced liver injury (SILI) is a major contributor to organ dysfunction and is closely associated with increased morbidity and mortality in septic patients. However, conventional liver biochemical indicators often fail to capture early mitochondrial and metabolic disturbances that precede overt hepatic dysfunction, limiting timely diagnosis and targeted intervention. Emerging evidence indicates that mitochondrial impairment plays a central role not only in bioenergetic failure but also in coordinating immune–metabolic dysregulation during sepsis. In this review, we critically synthesize current knowledge on mitochondrial injury in SILI, with a focus on its mechanistic links to cellular metabolic reprogramming, redox imbalance, and immune cell dysfunction. We highlight how these interconnected processes contribute to hepatocellular injury and disease progression. Furthermore, we summarize recently identified mitochondrial- and metabolism-associated biomarkers that show promise for earlier detection of liver dysfunction in sepsis. Rather than viewing mitochondrial dysfunction as an isolated event, we emphasize its role as a central mediator connecting metabolic stress and immune responses in the septic liver. This perspective provides a mechanistic basis for understanding disease heterogeneity and may facilitate the development of more precise diagnostic and therapeutic strategies.

## 1 Introduction

Sepsis is defined as life-threatening organ dysfunction caused by a dysregulated host response to infection (Singer et al., 2016). Despite advances in critical care, sepsis remains a major global health challenge, with an estimated 166 million cases and 21.4 million sepsis-related deaths worldwide in 2021 (Strnad et al., 2017; Rudd et al., 2020). Both the incidence and mortality of sepsis increase markedly with age, particularly among individuals aged over 70 years (Strnad et al., 2017; Rudd et al., 2020). The liver is a crucial target organ in sepsis, and hepatic dysfunction is strongly associated with increased mortality and poor clinical outcomes (Kobashi et al., 2013). Clinically, liver injury is commonly reflected by elevated circulating levels of bilirubin and bile acids, together with increased serum transaminases (Yan et al., 2014). However, these conventional indicators are often nonspecific and may not sensitively capture early mitochondrial and metabolic dysfunction during sepsis.

During sepsis, pathogen-associated molecular patterns (PAMPs) and damage-associated molecular patterns (DAMPs) activate innate immune signaling and trigger energy-intensive defense programs, including inflammatory responses and acute-phase protein synthesis (Li et al., 2024). These defense programs are highly energy-demanding and require substantial metabolic adaptation (Di Gioia et al., 2020). At the same time, reduced nutrient availability, systemic inflammatory stress, hypoxia, and microbial toxins profoundly impair mitochondrial function, resulting in an imbalance between energy demand and energy supply (Leitner et al., 2023). This metabolic imbalance is particularly detrimental in the liver, which orchestrates glucose and lipid metabolism, acute-phase protein synthesis, and detoxification. Accordingly, mitochondrial injury in this organ can have disproportionately broad systemic consequences during sepsis (Leitner et al., 2023; Horn and Tacke, 2024).

Although inflammatory mechanisms in sepsis have been extensively investigated, they do not fully explain the complex pathogenesis of SILI. In recent years, several reviews have separately summarized the roles of mitochondrial dysfunction, metabolic reprogramming, and hepatic injury during sepsis (Zheng et al., 2023; Horn and Tacke, 2024; Xu et al., 2025). These studies have substantially advanced our understanding of bioenergetic failure, inflammatory signaling, and metabolic adaptation in the liver. However, most existing reviews focus on individual mechanisms or systemic manifestations of sepsis and rarely address how mitochondrial dysfunction and immune metabolic remodeling interact to drive liver-specific injury. Furthermore, the emerging links among mitochondrial damage, immune-cell metabolic adaptation, biomarker development, and metabolism-targeted therapies have not been comprehensively synthesized within a unified framework for SILI. To our knowledge, no previous review has specifically integrated these interconnected processes into a liver-centered framework spanning pathogenesis, biomarker discovery, and therapeutic intervention in SILI.

Recent studies have increasingly highlighted extensive crosstalk between mitochondrial homeostasis and immune–metabolic regulation during sepsis, suggesting that these interconnected processes collectively contribute to the development of liver injury (Wang et al., 2021; Liu et al., 2022). However, these advances remain fragmented across different areas of research, and their implications for liver-specific injury during sepsis have not been systematically discussed within a single review. Accordingly, this review focuses on the current evidence linking mitochondrial dysfunction with immune–metabolic alterations in SILI, with the aim of clarifying their mechanistic interplay, summarizing emerging biomarkers, and discussing metabolism-based therapeutic strategies.

## 2 Literature search strategy

This review was primarily based on a literature search conducted in PubMed/MEDLINE between January 2020 and December 2025. Additional relevant articles published in 2026 were manually incorporated during manuscript revision to ensure that the review reflects the latest advances in the field. The search strategy combined Medical Subject Headings (MeSH) and free-text terms related to sepsis, mitochondria, immunometabolism, metabolic reprogramming, and liver injury, including “sepsis”, “sepsis-associated liver injury”, “SILI”, “mitochondria”, “mitochondrial dysfunction”, “mitochondrial transplantation”, “immunometabolism”, “metabolic reprogramming”, “liver injury”, “hepatic injury”, and “hepatocyte”. Additional topic-specific searches were performed during manuscript preparation to clarify particular mechanistic pathways discussed in the review. Original studies, experimental investigations, clinical studies, and relevant review articles published in English were considered. Conference abstracts, editorials, duplicate publications, and studies unrelated to the mitochondria-immunometabolism axis in sepsis-associated liver injury were excluded.

## 3 Mitochondrial dysfunction as a central driver of SILI

### 3.1 Mitochondrial structural collapse and bioenergetic–metabolic crisis

**Figure 1:**
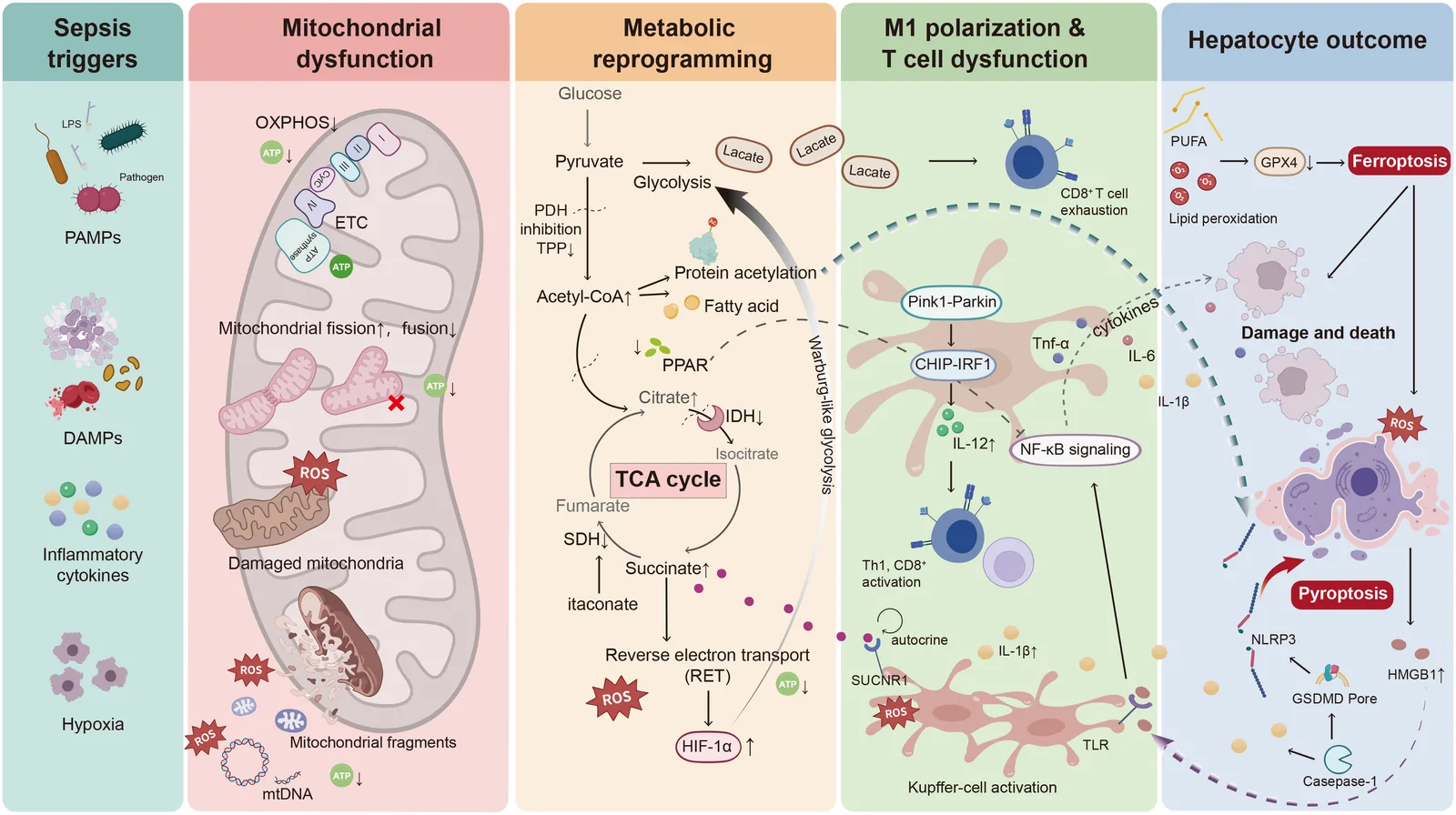
Mitochondria–immunometabolism axis in sepsis-induced liver injury. Sepsis triggers, including pathogen-associated molecular patterns (PAMPs), damage-associated molecular patterns (DAMPs), and hypoxia, initiate hepatocyte and immune cell stress. These signals induce mitochondrial dysfunction, characterized by impaired oxidative phosphorylation, ATP depletion, mitochondrial fragmentation, excessive ROS production, and mitochondrial damage-associated molecular signal release. Mitochondrial dysfunction drives metabolic reprogramming, including disruption of tricarboxylic acid (TCA) cycle activity, inhibition of pyruvate dehydrogenase, and succinate-associated redox imbalance, resulting in a metabolic shift toward glycolysis. Metabolic reprogramming promotes immune dysregulation, including macrophage M1 polarization, inflammasome activation, and T cell dysfunction, accompanied by increased production of pro-inflammatory cytokines. These immune alterations amplify inflammatory signaling and contribute to progressive hepatocellular injury. Ultimately, mitochondrial dysfunction, metabolic reprogramming, and immune activation converge to induce hepatocyte damage, including ferroptosis and pyroptosis. This framework illustrates a hierarchical cascade linking sepsis-induced mitochondrial dysfunction to metabolic and immune reprogramming, culminating in liver injury and cell death, and highlights mitochondria as a central regulatory hub in sepsis-induced liver injury.

Under septic stress, mitochondria undergo profound structural and functional deterioration that compromises coupled respiration and metabolic flexibility (**Figure 1**; Leitner et al., 2023). Because hepatocytes are highly enriched in mitochondria and depend on coordinated oxidative and biosynthetic metabolism, these abnormalities are particularly detrimental to hepatic homeostasis (Zheng et al., 2023). When exposed to septic insults, mitochondria undergo structural disorganization, disrupting the architecture required for coupled respiration and bioenergetic efficiency, particularly under conditions of nutrient deprivation (Horn and Tacke, 2024). These changes are characterized by enhanced cristae remodeling and excessive mitochondrial fragmentation, often accompanied by impaired mitochondrial fusion (Horn and Tacke, 2024). Functionally, this state is further associated with excessive generation of reactive oxygen species (ROS), particularly mitochondrial ROS (mtROS), depletion of adenosine triphosphate (ATP), sustained release of pro-apoptotic signals, impaired biosynthetic capacity, and defective mitophagy (Kozlov et al., 2011; Xu et al., 2025). Insufficient mitophagy clearance permits the persistence of dysfunctional mitochondria, thereby sustaining mtROS production and the release of mitochondria-derived danger signals (M S et al., 2015).

In addition, disruption of the balance between mitochondrial fission and fusion impairs mitochondrial function and increases susceptibility to cellular injury. Studies have shown that under septic conditions, mitochondrial fission mediated by dynamin-related protein 1 (DRP1) is enhanced, whereas mitochondrial fusion mediated by optic atrophy 1 (OPA1) and mitofusin 2 (MFN2) is reduced (Xu et al., 2025). OPA1 and MFN2 collectively preserve mitochondrial integrity by promoting membrane fusion and maintaining cristae organization. Altered expression or activity of DRP1, OPA1, and MFN2 disrupts fission–fusion homeostasis, resulting in excessive mitochondrial fragmentation and accumulation of damaged mitochondrial fragments (Xu et al., 2025). On the one hand, mitochondrial fragmentation compromises the functional mitochondrial network, thereby reducing oxidative phosphorylation efficiency and ATP synthesis (Youle and van der Bliek, 2012; Zhou et al., 2024). On the other hand, damaged mitochondrial components can function as damage-associated molecular patterns, thereby promoting inflammatory signaling and further ROS generation (Dela Cruz and Kang, 2018; Di Gioia et al., 2020). Collectively, these changes amplify inflammation and establish a vicious cycle.

Aberrant accumulation of metabolites and inflammatory mediators is another hallmark of septic mitochondrial dysfunction, including acetyl-CoA, citrate, succinate, and nitric oxide (NO). Acetyl-CoA provides the carbon input for the tricarboxylic acid (TCA) cycle, whereas citrate and succinate are key cycle intermediates; sustained accumulation of these metabolites in sepsis disrupts efficient aerobic ATP production. Acetyl-CoA is a central intermediate in carbohydrate and lipid metabolism, and its excessive accumulation may divert metabolic flux toward ketogenesis, thereby limiting the balanced supply of substrates to the electron transport chain (ETC) (Pietrocola et al., 2015). Excess citrate can inhibit glycolysis, reduce substrate availability for downstream aerobic oxidation, and exert feedback inhibition on key metabolic enzymes, thereby imposing coordinated suppression of glycolysis and TCA-cycle flux (Philippe and Hubert, 2016).

Succinate accumulation is closely linked to impaired succinate dehydrogenase (SDH) activity (Mills et al., 2016). SDH dysfunction is associated with ROS-mediated oxidative damage and disruption of mitochondrial membrane integrity (Lampropoulou et al., 2016). SDH also functions as Complex II of the mitochondrial electron transport chain and is embedded in the inner mitochondrial membrane (Lemarie et al., 2011; Lampropoulou et al., 2016). Hypoxia can directly impair electron transport, thereby reducing the efficiency of SDH-dependent electron transfer. In addition, the citrate-derived metabolite itaconate, together with elevated NO, can further inhibit SDH activity (Mills et al., 2016; Muniz-Santos et al., 2023; O’Carroll et al., 2024). Studies have shown that accumulated succinate can drive reverse electron transfer (RET) at Complex I, leading to excessive ROS production, aggravated damage to mitochondrial membranes and ETC proteins, and further impairment of oxidative phosphorylation (Chouchani et al., 2014; Mills et al., 2016). The accumulation of these metabolites ultimately contributes to ATP insufficiency and aggravates metabolic stress.

Nitric oxide (NO) production is also linked to succinate accumulation, particularly under conditions in which SDH-dependent oxidation of succinate to fumarate is impaired (Jha et al., 2015). Through the aspartate–argininosuccinate shunt, aspartate and citrulline are converted into argininosuccinate and subsequently arginine, which serves as the substrate for inducible nitric oxide synthase (iNOS)-mediated NO production (Jha et al., 2015). NO directly inhibits the electron transport chain (ETC), reduces mitochondrial membrane potential, and suppresses oxidative phosphorylation (OXPHOS), thereby decreasing ATP production (Everts et al., 2012; Bailey et al., 2019). In parallel, NO promotes further accumulation of TCA-cycle intermediates, reinforcing a positive feedback loop (Everts et al., 2012; Bailey et al., 2019).

### 3.2 Mechanisms linking mitochondrial dysfunction to immune reprogramming

Although multiple hepatic and circulating immune cell populations participate in this process, macrophages represent a central hub linking mitochondrial stress to inflammatory amplification. Mitochondrial dysfunction reshapes immune responses through at least three interconnected mechanisms: altered mitochondrial dynamics, release of mitochondria-derived danger signals, and accumulation of immunoregulatory metabolites. Macrophage polarization is closely linked to mitochondrial dynamics and metabolic state. Under septic conditions, mitochondrial dynamics shift toward fission rather than fusion (Zhang et al., 2022). Enhanced mitochondrial fission promotes cristae remodeling, reduces ETC efficiency, favors a shift toward aerobic glycolysis, and thereby facilitates M1 macrophage polarization. Fusion-promoting regulators, such as the outer mitochondrial membrane protein FAM73b, are downregulated upon LPS stimulation, leading to sustained mitochondrial fragmentation and enhanced M1 polarization (Park et al., 2015; Gao et al., 2017; Sloat et al., 2019). In addition, loss of FAM73b expression promotes Parkin activation. Beyond downregulating MFN1 and OPA1 through the PINK1–Parkin pathway to further promote mitochondrial fission, Parkin also regulates the CHIP–IRF1 axis, thereby enhancing IL-12 production (Gao et al., 2017). IL-12, which is secreted by antigen-presenting cells including macrophages, promotes Th1 differentiation, suppresses anti-inflammatory cytokines such as IL-10, and enhances the cytotoxic activity of natural killer (NK) cells and cytotoxic T lymphocytes (CTLs) (Elser et al., 2002; Trinchieri, 2003; Youle and van der Bliek, 2012).

However, it should also be noted that DRP1 upregulation and enhanced mitochondrial fission do not uniformly promote NLRP3 inflammasome-dependent caspase-1 activation and IL-1β secretion; in some contexts, these processes may instead facilitate the early clearance of damaged mitochondria (Gao et al., 2017; Sloat et al., 2019). Notably, persistent TLR4 activation during sepsis upregulates the expression of *Nlrp3* and *Il1b* through the NF-κB pathway (Zhang et al., 2025). At the same time, extracellular ATP activates P2X7 signaling, while ERK pathway activation further synergistically amplifies the NLRP3 response (Guggemos et al., 2024). Once activated by leaked mitochondrial components, the NLRP3 inflammasome can further promote mtROS generation, mtDNA release, and additional mitochondrial damage (Zhang et al., 2025). In this context, mitochondrial dysfunction cooperates with canonical inflammasome priming and activation signals, rather than acting as an isolated trigger. These signals may outweigh the effect of DRP1 alone, suggesting that enhanced NLRP3 activation remains a dominant driver of inflammatory amplification (Park et al., 2015).

Metabolic imbalance is a major driver of immune dysregulation. Beyond serving as metabolic intermediates, these molecules also function as signaling cues that shape inflammatory gene expression and immune-cell fate. In addition to mtROS, excessive acetyl-CoA may promote NLRP3 inflammasome activation through protein acetylation-dependent mechanisms. Specifically, increased acetyl-CoA availability facilitates direct acetylation of NLRP3, thereby enhancing its oligomerization and assembly (He et al., 2020). Concurrently, mitochondrial hyperacetylation may promote the aggregation of apoptosis-associated speck-like protein containing a CARD (ASC), further facilitating inflammasome complex formation and IL-1β maturation (Misawa et al., 2013). Moreover, acetyl-CoA serves as an essential acetyl donor for histone acetylation, thereby enhancing the transcription of pro-inflammatory genes, including tumor necrosis factor-α (TNF-α) and interleukin-6 (IL-6), which amplifies inflammatory responses (Langston et al., 2019). Citrate can also function as a metabolic switch in immune-cell differentiation, a process that is facilitated by LPS-induced downregulation of isocitrate dehydrogenase (IDH) (Yang et al., 2024). In addition, citrate promotes M1 macrophage polarization, at least in part by enhancing iNOS expression and NO production (Boscá et al., 2005).

Finally, metabolic intermediates, particularly succinate, function as immunometabolic signals that link mitochondrial dysfunction to inflammatory activation in immune cells. Succinate promotes mtROS generation through SDH-dependent reverse electron transfer (RET), which stabilizes HIF-1α and thereby enhances Warburg-like metabolic reprogramming and IL-1β expression (Koivunen et al., 2007; Mills et al., 2016). In addition, direct inhibition of SDH by itaconate, a citrate-derived metabolite induced during inflammatory signaling, together with activation of the LPS-induced aspartate–argininosuccinate shunt, further limits SDH-dependent flux and contributes to succinate accumulation (Lampropoulou et al., 2016). Collectively, these metabolite-driven signals amplify inflammasome-related inflammatory responses in a context-dependent manner (Koivunen et al., 2007; Lampropoulou et al., 2016; Mills et al., 2016). 

In addition to promoting pro-inflammatory macrophage activation, mitochondrial metabolic failure may also reshape adaptive immune responses. Elevated lactate levels are common during sepsis progression and are strongly associated with sepsis-related mortality (Singer et al., 2016). This phenomenon has been attributed, at least in part, to dysfunction of the pyruvate dehydrogenase complex (PDC), particularly increased activity of pyruvate dehydrogenase kinase (PDK), which enhances PDH phosphorylation and thereby inhibits its activity (Nuyttens et al., 2025). Further studies suggest that deficiency of the essential cofactor thiamine pyrophosphate (TPP) may further impair this complex, preventing pyruvate from being converted into acetyl-CoA for entry into the tricarboxylic acid (TCA) cycle and thereby increasing cellular dependence on glycolysis (Nuyttens et al., 2025). This shift represents a rapid adaptive response to energetic stress, but at the cost of excessive lactate production (Zhang et al., 2019). Although glutamic pyruvate transaminase 2 (GPT2) can partially compensate by catalyzing the transamination of pyruvate with glutamate to generate alanine and α-ketoglutarate, which can enter the TCA cycle and support oxaloacetate (OAA) replenishment, this compensatory pathway is clearly insufficient to offset pyruvate accumulation caused by blockade of the major PDH-dependent metabolic route (Nuyttens et al., 2024; Nuyttens et al., 2025). Beyond innate inflammatory amplification, mitochondrial metabolic failure may also reshape broader immunometabolic programs affecting adaptive immunity and inflammatory resolution. High lactate levels may contribute not only to numerical decline and functional exhaustion of CD8+ T cells but also to impaired T cell metabolic adaptation, thereby weakening effector function and antimicrobial immunity during later stages of sepsis (Imai et al., 2019).

Conversely, intact mitochondrial lipid-handling pathways may also support the resolution phase of inflammation. Oxylipins, which are oxidized polyunsaturated fatty acids generated by cyclooxygenases (COX), lipoxygenases (LOX), and cytochrome P450 enzymes (CYP), act as potent inflammatory mediators and regulate immune-cell functions such as chemotaxis, phagocytosis, and cytokine secretion through G protein-coupled receptors (GPCRs) (Simmons et al., 2004; Capdevila et al., 2007; Serhan et al., 2008). Timely clearance of these mediators is therefore necessary to prevent excessive inflammatory amplification and tissue injury. Studies have shown that under LPS/IFN-γ stimulation, macrophages upregulate carnitine palmitoyltransferase 1 (CPT1), enabling lipoxin handling through a pathway analogous to that used for long-chain fatty acids, including acylcarnitine formation and mitochondrial transport for β-oxidation (Slatter et al., 2016). The resulting degradation products no longer effectively bind GPCRs and therefore lose much of their pro-inflammatory activity (Slatter et al., 2016). Notably, β-oxidation of lipoxins appears to contribute primarily to inflammatory resolution rather than ATP generation (Misheva et al., 2022a). Their clearance restrains excessive macrophage activation, helps maintain the balance between pro-inflammatory and anti-inflammatory responses, reduces macrophage secretion of TNF-α and IL-6, and limits additional ROS production (Misheva et al., 2022a).

## 4 Cell-specific immunometabolic reprogramming in SILI

### 4.1 Hepatocellular metabolic reprogramming and ferroptotic vulnerability

Building on these upstream mitochondrial and immunoregulatory mechanisms, the following section focuses on how such reprogramming is executed in specific hepatic cell compartments. During the acute phase of sepsis, hyperglycemia is commonly observed. This is manifested in at least two ways. First, glucocorticoid receptor (GR) signaling suppresses glucose transporter 4 (GLUT4)-mediated glucose utilization and is associated with upregulation of gluconeogenic gene expression (Bose et al., 2016). As a result, glucose utilization is reduced, while endogenous glucose production is increased. Second, many patients develop insulin resistance, which further impairs glucose uptake and shifts energy metabolism toward hepatic glycogen consumption and lipid mobilization (Muniz-Santos et al., 2023). Under these conditions, hepatic glycogen stores are rapidly depleted, making energy insufficiency increasingly likely (Bose et al., 2016; Muniz-Santos et al., 2023). As glucose utilization becomes progressively impaired, hepatocytes increasingly rely on lipid substrates as an alternative energy source; however, this compensatory shift is often inefficient under septic conditions (Muniz-Santos et al., 2023).

Compensatory energy supply through lipid metabolism is often inefficient during sepsis. Evidence suggests that as sepsis reduces glucose-utilization efficiency, systemic energy metabolism shifts toward lipid mobilization, resulting in marked elevation of circulating free fatty acid (FFA) levels (Oh et al., 2022). After entering the liver, FFAs are converted into acylcarnitines in a CPT1-dependent manner for transport into mitochondria. In addition, inflammatory conditions increase the demand for oxylipin clearance (Obici et al., 2003; Misheva et al., 2022b). This process may consume available carnitine and thereby limit the mitochondrial transport of remaining long-chain fatty acids (LCFAs) (Obici et al., 2003; Misheva et al., 2022b). Meanwhile, acetyl-CoA accumulation can further redirect metabolic flux toward lipid synthesis. Together, these alterations promote hepatic lipid accumulation, induce lipotoxicity, and severely compromise hepatocyte viability and function (Oh et al., 2022).

These metabolic abnormalities are reinforced by transcriptional reprogramming that suppresses hepatocellular oxidative metabolism and lipid-handling capacity. Several metabolism-related transcription factors are also critically involved, among which the nuclear receptor peroxisome proliferator-activated receptor α (PPARα) is particularly important. PPARα primarily functions through ligand-dependent transcriptional activation of genes encoding lipid transport proteins, acetyl-CoA synthases, and enzymes involved in β-oxidation and ketogenesis (Polvani et al., 2016). It is one of the key sensors that helps maintain metabolic homeostasis during nutrient deprivation and energy deficiency (Van Dender et al., 2024). In addition, PPARα can suppress NF-κB signaling and reduce the production of pro-inflammatory mediators in inflammatory conditions (Romics et al., 2004). Studies have shown that PPARα is frequently suppressed during the early stage of sepsis, leading to impaired fatty acid metabolism and excessive accumulation of free fatty acids (FFAs) (Van Dender et al., 2024).

Another key regulator is hepatocyte nuclear factor 4 alpha (HNF4α), a nuclear receptor that serves as an upstream regulator of lipid metabolism. Abnormally reduced HNF4α activity can also downregulate PPARα by weakening binding to the DR1 motif within the PPARα promoter (Van Dender et al., 2024). Moreover, reduced HNF4α activity is associated with decreased H3K27ac levels at enhancers of metabolic genes, while acetylation increases at inflammation-related loci (Englert et al., 2015; Van Dender et al., 2024). In addition, partial loss of HNF4α significantly disrupts IL-6-induced acute-phase response (APR) gene and protein expression (Van Dender et al., 2024). This impairs hepatocyte regeneration and is associated with increased sepsis lethality, hepatic steatosis, and organ damage (Van Dender et al., 2024). Notably, under physiological conditions, HNF4α also restrains hepatocyte proliferation (Huck et al., 2019). However, in severe acute liver injury caused by drugs, infection, or virulence factors—especially during sepsis—an overwhelming inflammatory response induces widespread parenchymal cell death, and downregulation of HNF4α may further aggravate the already fragile metabolic state (Van Dender et al., 2025). Although this change may trigger compensatory regenerative signaling to some extent, hepatocyte regeneration may still fail to keep pace with the rate of tissue injury. Failure to fully replace lost hepatocytes may ultimately culminate in liver failure.

Ferroptosis represents a key immunometabolic interface linking mitochondrial dysfunction in hepatocytes with inflammatory activation in immune cells. As a mitochondria-rich organ, the liver is highly vulnerable to septic metabolic stress. Because mitochondria are major sites of ROS generation, impaired oxidative phosphorylation and electron transport chain dysfunction during sepsis promote excessive mtROS production and disrupt redox homeostasis. At the same time, mitochondrial dysfunction compromises ATP-dependent antioxidant defenses and perturbs iron–sulfur cluster biogenesis, contributing to intracellular iron dysregulation and increased susceptibility to lipid peroxidation. These mitochondrial events mechanistically link bioenergetic failure to ferroptotic vulnerability in hepatocytes. Beyond disturbances in glucose and lipid utilization, septic metabolic reprogramming also disrupts hepatocellular redox balance and bile acid homeostasis. Inflammatory signaling induced by septic factors, including TNF-α and IL-6, together with mitochondrial dysfunction and suppression of the nuclear receptors PPARα and FXR, disrupts bile acid homeostasis and promotes accumulation of toxic bile acids such as deoxycholic acid (Thomas, 2017). These changes enhance oxidative stress and ROS generation. At the same time, iron homeostasis becomes dysregulated, partly through pro-inflammatory upregulation of iron transporters such as transferrin receptor, which increases intracellular iron uptake (Mertens et al., 2025). In addition, labile Fe²^+^ accumulates as iron-storage proteins such as ferritin are degraded (Weis et al., 2017). This promotes Fenton chemistry, which drives peroxidation of membrane polyunsaturated fatty acids (PUFAs) that are enriched under conditions of dysregulated lipid metabolism. As a result, lipid peroxidation products such as malondialdehyde (MDA) and 4-hydroxynonenal (4-HNE) accumulate to high levels (Rodrigues et al., 1998b; Rodrigues et al., 1998a).

Meanwhile, persistent mitochondrial ROS generation, impaired NADPH regeneration, and septic inflammatory signaling contribute to depletion of reduced glutathione (GSH) and suppression of glutathione peroxidase 4 (GPX4), a key antioxidant enzyme responsible for detoxifying phospholipid hydroperoxides (Van Coillie et al., 2022). At the same time, the Nrf2 pathway, which regulates antioxidant gene expression, is suppressed in sepsis, preventing timely clearance of hepatic lipid peroxides and allowing their progressive accumulation (Thimmulappa et al., 2006; Van Coillie et al., 2022). Consequently, lipid peroxidation products such as MDA and 4-HNE directly damage membrane phospholipids, compromise membrane integrity, promote lytic cell death, and exacerbate organ injury (N et al., 2020; Liu et al., 2023). Furthermore, DAMPs such as HMGB1 released during ferroptosis can activate the NLRP3 inflammasome and TLR signaling pathways, thereby forming a self-reinforcing oxidative stress–inflammation–ferroptosis loop (Liu and Liang, 2025). Collectively, these changes position ferroptosis not merely as a downstream consequence of hepatocellular metabolic collapse, but as a key immunometabolic amplifier that links mitochondrial dysfunction, redox imbalance, iron dysregulation, and inflammatory signaling, thereby reinforcing immune–metabolic crosstalk in SILI.

### 4.2 Kupffer cell–hepatocyte metabolic crosstalk in SILI

Kupffer cells serve as a key immunometabolic amplification hub in the liver, integrating circulating metabolic signals with local inflammatory and cell death pathways. Evidence suggests that liver-resident macrophages, namely Kupffer cells (KCs), sense accumulated succinate through succinate receptor 1 (SUCNR1), thereby inducing IL-1β synthesis and release (Nuyttens et al., 2025; Liu et al., 2026). The resulting IL-1β-dependent inflammatory signaling promotes inflammasome activation and contributes to hepatocellular injury. Inflammasome activation subsequently induces caspase-1 activation and gasdermin D (GSDMD) cleavage, leading to membrane pore formation (Vande Walle and Lamkanfi, 2024).

Through these pores, DAMPs such as HMGB1 are released, which exacerbate liver inflammation and secondary tissue injury through TLR-dependent signaling pathways (Tannahill et al., 2013). Concurrently, persistent succinate signaling and IL-1β production in the hepatic microenvironment impair hepatocellular antioxidant defenses and promote oxidative stress (Baksh and Finley, 2021; Nuyttens et al., 2025). Together with iron overload, these changes promote lipid peroxidation and synergistically contribute to GPX4 inhibition (Liu and Liang, 2025). These processes ultimately promote ferroptosis in hepatocytes. This local immunometabolic circuit may be further intensified by the gut–liver axis. Importantly, the liver is uniquely exposed to gut-derived metabolites through the portal circulation. As a result, microbiota-derived succinate can enter the liver directly via the portal vein, continuously exposing hepatocytes to high local succinate levels and potentially aggravating injury (Xu et al., 2023; Y et al., 2024). Beyond receptor-mediated inflammatory signaling, excess succinate may also directly perturb intracellular metabolism through abnormal protein succinylation (Du et al., 2011). High levels of protein succinylation, including that of malate dehydrogenase and GAPDH, may also arise as a consequence of inactivation of sirtuin-family lysine desuccinylases, thereby impairing the function of key metabolic enzymes (Du et al., 2011; Tannahill et al., 2013).

Taken together, these findings indicate that immunometabolic reprogramming in SILI is not restricted to hepatocytes but extends to nonparenchymal-cell-mediated inflammatory crosstalk. Metabolic stress in hepatocytes, together with succinate-driven activation of Kupffer cells and downstream inflammatory amplification, establishes a self-reinforcing immunometabolic circuit that drives the progression from adaptive dysfunction to irreversible liver injury.

## 5 Emerging biomarkers and diagnostic approaches

**Figure 2:**
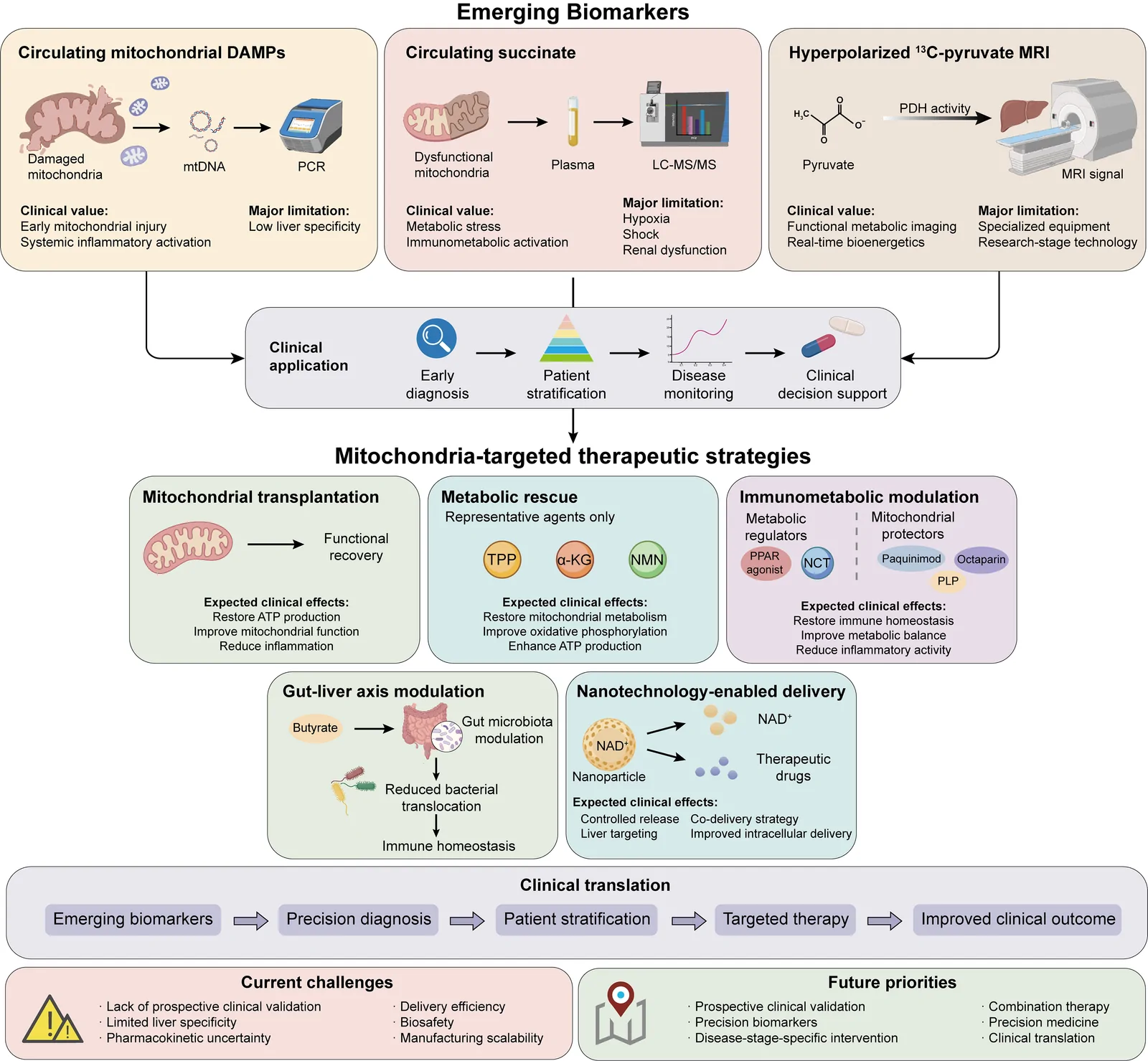
Emerging mitochondrial biomarkers and mitochondria-targeted therapeutic strategies for clinical translation. Emerging biomarkers of mitochondrial dysfunction include circulating mitochondrial DAMPs (e.g., mtDNA), metabolic intermediates (e.g., succinate), and functional metabolic imaging using hyperpolarized ^13C-pyruvate MRI. These biomarkers reflect mitochondrial injury and metabolic reprogramming and enable early diagnosis, patient stratification, disease monitoring, and clinical decision support. Mitochondria-targeted therapeutic strategies include mitochondrial transplantation, metabolic rescue, immunometabolic modulation, gut–liver axis regulation, and nanotechnology-enabled delivery systems. Collectively, these approaches link mitochondrial dysfunction to metabolic and immune dysregulation, providing a translational framework from biomarker identification to targeted intervention and improved clinical outcomes in sepsis-induced liver injury.

Given the limited sensitivity and mechanistic specificity of conventional liver biochemical tests in sepsis, biomarkers reflecting mitochondrial injury and metabolic dysfunction may provide complementary information for the early identification and pathophysiological stratification of SILI (**Figure 2**). However, none of the currently available candidates has been prospectively validated in SILI-specific clinical cohorts, and their diagnostic utility remains largely exploratory. Future studies are therefore required to determine their sensitivity, specificity, feasibility, and prognostic value in critically ill patients.

### 5.1 Circulating mitochondrial DAMPs

Mitochondrial DAMPs are readily released into the circulation following mitochondrial injury and mainly include formyl peptide-containing protein fragments and mitochondrial DNA (mtDNA) (D’Amico et al., 2017). Once released, formyl peptides and mitochondrial DNA bind to formyl peptide receptor 1 (FPR1) and Toll-like receptor 9 (TLR9), respectively, thereby promoting neutrophil migration and degranulation through Ca2^+^ flux and MAPK phosphorylation (Zhang et al., 2010a; Dela Cruz and Kang, 2018). Accordingly, molecular assays for plasma nucleic-acid fragments, such as polymerase chain reaction (PCR), together with antibody-based detection of formyl peptides, may provide useful diagnostic information (He et al., 2002). Although these markers are not liver-specific, they may provide early insight into systemic mitochondrial injury and inflammatory activation during sepsis. They may be most useful when interpreted in combination with conventional liver-function indices and other metabolic markers, rather than as standalone indicators of SILI.

Clinical studies have demonstrated marked elevations of circulating mtDNA in trauma-associated systemic inflammatory responses, supporting its potential role as an indicator of tissue injury and innate immune activation (Zhang et al., 2010b). In a study of critically ill patients with sepsis, serum mitochondrial-encoded NADH dehydrogenase 6 (MT-ND6) together with Annexin A1 was identified as a novel biomarker panel for mortality prediction. It highlights the clinical relevance advantage of combining circulating mitochondrial-derived molecules with other markers, compared to conventional inflammatory markers (Zhou et al., 2024). The finding further supports the concept that mtDAMP-associated biomarkers reflect the severity of mitochondrial injury and systemic inflammation. However, whether these biomarkers possess sufficient diagnostic specificity for SILI remains to be determined. Moreover, mtDAMP concentrations may be influenced by trauma, ischemia-reperfusion injury, malignancy, and dysfunction of other organs, thereby limiting liver specificity. From a practical perspective, PCR-based quantification of circulating mtDNA is technically feasible in tertiary-care laboratories but is not routinely available for real-time decision-making in most ICU settings. Compared with conventional liver biochemical markers, mtDAMPs may provide complementary mechanistic information regarding mitochondrial injury and systemic inflammatory activation; nevertheless, their incremental diagnostic value for SILI remains to be established.

### 5.2 Circulating succinate as a candidate metabolic biomarker

As a central metabolic organ, the liver is particularly susceptible to mitochondrial dysfunction, which directly contributes to succinate accumulation. On the one hand, excess succinate promotes ROS generation through reverse electron transfer (RET) and impairs ATP production. On the other hand, succinate activates Kupffer cells through SUCNR1 signaling to induce IL-1β production, thereby exacerbating injury in neighboring hepatocytes and promoting a self-amplifying inflammatory feedback loop (Tannahill et al., 2013). Because succinate can be rapidly released into the circulation and is enriched in mitochondria-dependent metabolic tissues, it may serve as a potentially informative indicator of hepatic mitochondrial dysfunction. This dual role as both a pathogenic metabolite and a measurable circulating signal makes succinate particularly attractive for mechanism-based biomarker development. Targeted mass spectrometry-based quantification of succinate in plasma or serum may therefore represent a promising translational diagnostic approach (Cui et al., 2021).

Several clinical metabolomic studies have demonstrated that plasma succinate concentrations possess diagnostic discriminatory value in inflammatory cardiovascular diseases, including the ability to distinguish aortic aneurysm and dissection from healthy controls and other acute cardiovascular conditions (Cui et al., 2021). Nevertheless, no studies have prospectively evaluated succinate as a biomarker of SILI, and clinically validated sensitivity and specificity thresholds are lacking. Furthermore, circulating succinate concentrations may be influenced by systemic hypoxia, shock-related tissue ischemia, renal dysfunction, and mitochondrial injury in extrahepatic tissues, all of which may confound interpretation in critically ill patients. Although targeted mass spectrometry assays are increasingly available in research settings, they remain insufficiently standardized for routine ICU implementation. Compared with conventional liver biochemical markers, succinate may better reflect mitochondrial metabolic stress and immunometabolic activation; however, its utility as an early biomarker of SILI remains hypothetical and requires prospective validation.

### 5.3 Noninvasive assessment of hepatic PDH activity

PDH activity is frequently inhibited under septic stress and may serve as a functional readout of the shift from mitochondrial oxidation to compensatory glycolysis. To assess PDH activity safely and effectively, hyperpolarized ¹³C-pyruvate MRI has emerged as a promising noninvasive imaging approach (Hu et al., 2011). This method may be particularly valuable for translational studies aimed at identifying early hepatocellular bioenergetic failure before overt biochemical decompensation becomes evident. This technique is based on dynamic nuclear polarization (DNP), which enhances the signal of ¹³C-pyruvate by more than 10,000-fold and enables real-time detection of metabolic intermediates (Sharma et al., 2019). Following administration of hyperpolarized [1-¹³C] pyruvate, ¹³C-labeled metabolites such as lactate, alanine, malate, aspartate, and bicarbonate can be tracked with second-scale temporal resolution using magnetic resonance spectroscopy (MRS) (Keshari et al., 2013). This technique enables real-time monitoring of metabolic processes and ultimately allows noninvasive quantification of hepatic PDH activity. However, although this technique has been validated in the heart, its application in the liver is complicated by the presence of alternative bicarbonate-producing pathways, including phosphoenolpyruvate carboxykinase (PEPCK)- and pyruvate carboxylase (PC)-related pathways (Sharma et al., 2019). Notably, PC together with PEPCK may still dominate the ¹³C-bicarbonate signal even when PDH flux is low. Therefore, the potential confounding effects of PC and PEPCK should be considered when this technique is used to assess hepatic PDH activity (Sharma et al., 2019).

Preclinical studies have demonstrated that hyperpolarized ¹³C-pyruvate MRI can sensitively detect alterations in hepatic PDH flux and real-time pyruvate oxidation, supporting its utility for assessing mitochondrial bioenergetics (Sharma et al., 2019). However, no studies have established the diagnostic sensitivity or specificity of this technique for SILI. In addition, interpretation may be influenced by systemic metabolic alterations and hepatic perfusion abnormalities. It may also be helpful to combine this approach with the lactate/alanine ratio, which reflects cytosolic NADH/NAD^+^ status, and the malate C1/C4 ratio, which provides information on TCA-cycle kinetics (Merritt et al., 2011; Sharma et al., 2019). Although hyperpolarized ¹³C-pyruvate MRI provides unique functional information that cannot be obtained from conventional biochemical markers, its requirement for specialized hyperpolarization equipment, dedicated imaging infrastructure, and expert data analysis substantially limits feasibility in the ICU setting. Future biomarker development will likely depend on multimodal panels that integrate mitochondrial injury markers, metabolic readouts, and conventional clinical indices. At present, however, this method is more appropriately regarded as an advanced research and translational imaging tool than a routine clinical diagnostic assay.

Collectively, these emerging biomarkers provide promising mechanistic insights into mitochondrial injury and immunometabolic dysfunction in SILI. In addition, a study demonstrated that inflammatory biomarkers, including S100A8/A9 and resistin, served as independent predictors of mortality in critically ill patients with sepsis, further supporting the notion that biomarker panels integrating mitochondrial injury, metabolic dysfunction, and systemic inflammatory activation may offer greater clinical value than any single biomarker alone (Chen et al., 2025). Future studies should focus on prospective clinical validation, standardization of measurement approaches, and determination of their incremental value beyond conventional liver biochemical markers.

## 6 Targeting immunometabolic reprogramming in SILI

### 6.1 Mitochondrial transplantation

Given the marked loss of mitochondrial integrity and insufficient functional mitochondrial capacity during sepsis, direct transplantation of healthy mitochondria has emerged as a potential therapeutic strategy (**Table 1**). Investigators compared mitochondria isolated from L6 skeletal muscle cells, clone 9 hepatocyte cells, and umbilical cord-derived mesenchymal stem cells using LPS-stimulated THP-1 cells and a polymicrobial fecal-slurry rat model of sepsis (Kim et al., 2023). In experimental sepsis models, L6- and clone 9-derived mitochondria exhibited greater ATP synthetic capacity than MSC-derived mitochondria, whereas L6-derived mitochondria produced the greatest improvement in mitochondrial respiration. Mitochondrial transplantation also exerted bidirectional immunomodulatory effects and, in the rat sepsis model, L6-derived mitochondria improved survival and reduced plasma ALT and creatinine levels (Kim et al., 2023).

**Table 1:** Therapeutic approaches targeting mitochondrial dysfunction and immunometabolic pathways in sepsis.

Classification	Mechanisms of targeting	Drug/Treatment	Evidence of therapeutic efficacy	Experimental stage	Advantages and limitations	References
Direct mitochondrial supplementation	Direct supplementation of functional mitochondria to restore OXPHOS and ATP production	Mitochondrial transplantation	Improved mitochondrial respiration (OCR↑, ATP↑); reduced organ injury (ALT↓, creatinine↓); enhanced survival in sepsis models	Preclinical	Directly rescues bioenergetic failure and modulates immune responses; Source variability and delivery challenges limit clinical translation	(Merritt et al., 2011)
Immunometabolic modulator	PPARα activation restoring fatty acid β-oxidation and metabolic homeostasis	Pemafibrate	Reduced FFA and hepatic lipid accumulation; improved liver/kidney function (ALT/AST↓); decreased IL-6 and bacterial load	Preclinical	Dual metabolic and anti-inflammatory effects; Limited clinical evidence; efficacy depends on timing	(Kim et al., 2023)
	Activation of HNF4α–PPARα axis restoring ketogenesis and acute phase response	N-trans-caffeoyltyramine (NCT)	Improved survival; reduced ALT/AST and LDH; normalized lipid metabolism	Preclinical	Targets host metabolic tolerance independent of pathogen load; Lack of clinical validation; timing-sensitive	(Van Dender et al., 2024)
	Targets mitochondrial cardiolipin–GSDMD axis to inhibit pyroptosis and inflammation	Octaparin	Improved survival; reduced cytokines (TNF-α, IL-6); restored mitochondrial function and reduced ROS	Preclinical	Multi-target (mitochondria+inflammation+coagulation); Bleeding risk; no clinical data	(Zhang et al., 2025)
	Combined delivery of α-KG and spermine to restore TCA cycle function and promote anti-inflammatory (M2-like) macrophage polarization	spermine(encap)paKG MPs	Enhanced mitochondrial respiration; reduced pro-inflammatory macrophage phenotype (CD80/CD86↓); improved liver injury markers (ALT↓)	Preclinical	Synergistic regulation of metabolism and immune response with targeted delivery; Complex formulation; dose-dependent effects and lack of clinical validation	(Chen et al., 2025)
	S100A9 inhibition and AMPK activation improving mitochondrial metabolism	Paquinimod (Paq)	Reduced liver injury (ALT/AST↓); decreased inflammation and ROS; improved ATP production	Preclinical (Phase II in other diseases)	Multi-pathway regulation (metabolism+inflammation); Long-term safety unclear	(You et al., 2025)
	NAD^+^ precursor restoring mitochondrial function via SIRT pathways	nicotinamide mononucleotide (NMN)	Reduced liver injury and pyroptosis; improved ATP levels and bacterial clearance	Preclinical (clinical trials in other indications)	Good safety profile; Short half-life; bioavailability issues	(Van Wyngene et al., 2020; Lee et al., 2022; Inamdar et al., 2023)
	Inhibits mtDNA-mediated cGAS–STING activation	pyridoxal 5’-phosphate (PLP)	Reduced mtROS and pro-inflammatory cytokines; improved survival in sepsis models	Preclinical (Phase II in Coronary artery bypass graft)	Targets upstream inflammatory signaling; Potential off-target metabolic effects	(Li et al., 2023; Zhang et al., 2023)
	Restores PDH activity and pyruvate oxidation	TPP-plus-glucose	Reduced lactate; improved mitochondrial respiration; decreased mortality	Preclinical	Simple and cost-effective; Limited clinical validation	(Nuyttens et al., 2025)
Novel delivery system	Nanoparticle-mediated NAD^+^ delivery enhancing mitochondrial and immune homeostasis	NAD(H)-loaded NPs	Improved survival; reduced cytokines; restored ATP; enhanced antibacterial effect	Preclinical	Targeted delivery with dual therapeutic effects; High cost; unclear long-term safety	(Mao et al., 2024)

These findings suggest that the therapeutic effects of mitochondrial transplantation may depend on the cellular origin and bioenergetic capacity of donor mitochondria. However, the current evidence remains preliminary and does not establish a clinically applicable mitochondrial source for human SILI, because donor-source selection, species compatibility, isolation and quality-control standards, and scalability for human use remain unresolved. Other key translational issues include the efficiency of mitochondrial uptake by hepatocytes and immune cells *in vivo*, biodistribution after systemic administration, persistence and functional integration of transplanted mitochondria, potential immunogenicity, and long-term biosafety. Moreover, it remains uncertain whether the reduction in ALT observed in experimental sepsis models reflects direct protection against SILI or secondary improvement resulting from attenuation of systemic sepsis. To date, no clinical trials have evaluated mitochondrial transplantation in patients with sepsis or SILI. Therefore, mitochondrial transplantation should currently be regarded as a promising but highly experimental immunometabolic intervention, and its clinical applicability will remain uncertain until phase I/II studies establish safety, feasibility, pharmacokinetic profiles, biodistribution, and disease-specific efficacy in critically ill patients. Standardization of donor mitochondrial sources, optimization of delivery efficiency, and establishment of long-term safety profiles therefore remain the principal bottlenecks limiting clinical translation.

### 6.2 Metabolic and immunometabolic interventions

In the context of immune dysfunction driven by mitochondrial dysfunction, nutrient deprivation, and stress, several metabolically targeted agents may help mitigate septic liver injury or slow its progression. Their therapeutic effects may extend beyond restoration of mitochondrial oxidative phosphorylation and ATP synthesis to include improved glucose utilization, corrected lipid metabolism, and reduced histopathological tissue damage. These benefits may manifest as reduced apoptosis and necrosis, decreased immune-cell infiltration, suppression of pro-inflammatory phenotypes, and enhancement of anti-inflammatory immune responses. Additionally, reductions in organ-injury markers and pro-inflammatory cytokine levels are generally regarded as encouraging therapeutic outcomes.

Analogous to mitochondrial replacement, direct supplementation of metabolites depleted by metabolic blockade may also be beneficial. A first category of intervention aims to replenish metabolites or cofactors depleted by septic metabolic blockade. For example, thiamine pyrophosphate (TPP), an essential coenzyme of the pyruvate dehydrogenase complex (PDC), may help restore PDC function (Nuyttens et al., 2025). This promotes oxidation of pyruvate to acetyl-CoA and, when combined with adequate glucose supply, may synergistically enhance TCA-cycle activity and energy production (Nuyttens et al., 2025). In addition, supplementation with α-ketoglutarate (α-KG) may alleviate energetic stress by replenishing TCA-cycle intermediates (Inamdar et al., 2023). Alternatively, agents that target lipid toxicity may provide therapeutic benefit. A second strategy focuses on restoring hepatocellular lipid handling and transcriptional control of oxidative metabolism. These include pemafibrate, a PPARα activator, and N-trans-caffeoyltyramine (NCT), an activator of HNF4α, both of which may help limit excessive hepatic FFA accumulation (Van Wyngene et al., 2020; Inamdar et al., 2023; Van Dender et al., 2024). Changes in the tissue microenvironment and metabolite availability can strongly influence immune-cell polarization and the phagocytic function of macrophages and other immune cells. Delivery of α-KG to restore TCA-cycle metabolism may help support an M2-like macrophage phenotype. In addition, nicotinamide mononucleotide (NMN), a precursor of NAD^+^, may enhance the bactericidal capacity of macrophages by improving mitochondrial function (Li et al., 2023; You et al., 2025).

Additionally, strategies aimed at restoring mitochondrial structure and intrinsic function may also be beneficial. Beyond metabolic supplementation, some interventions aim to directly restore mitochondrial architecture and suppress mitochondria-driven inflammatory amplification. For example, Octaparin has been reported to restore mitochondrial cristae structure, whereas paquinimod (Paq) enhances complex I/II oxidative phosphorylation and electron transport system (ETS) activity (Zhang et al., 2023). These interventions may reduce mitochondrial fragmentation, limit mtDNA release and ROS generation, suppress NF-κB-mediated pro-inflammatory cytokine production, and enhance AMPK signaling and GLUT4 expression (Zhang et al., 2023; Zhang et al., 2025). Notably, during overwhelming inflammatory responses, accumulation of mitochondrial ROS and release of mtDNA from fragmented mitochondria can strongly amplify inflammation. Pyridoxal 5′-phosphate (PLP), an inhibitor of the pyrimidine nucleotide carrier SLC25A33, can inhibit mtROS production and alter the metabolic reprogramming of macrophages (MEND-CABG II Investigators et al., 2008; Kim et al., 2025). It may also directly suppress activation of the cGAS–STING pathway, which senses cytosolic DNA fragments, thereby reducing mtDNA replication during M1 macrophage polarization and limiting mtDNA release through voltage-dependent anion channel 1 (VDAC1) oligomerization (MEND-CABG II Investigators et al., 2008; Kim et al., 2025). Together with inhibition of the NF-κB pathway, these effects may reduce inflammatory mediator production, interrupt inflammatory positive-feedback signaling, and improve survival in experimental models (Kim et al., 2025).

Finally, sepsis is often accompanied by gut microbiota dysbiosis, barrier disruption, and bacterial translocation, making microbiota-targeted interventions particularly important. Because septic immunometabolic dysfunction is shaped in part by the gut–liver axis, microbiota-directed interventions may offer complementary benefits. In this context, butyrate enema therapy may represent a potential intervention. By activating PPAR-γ in the colonic epithelium, butyrate can reduce iNOS expression and thereby decrease nitrate production (Lefere et al., 2020). This may inhibit overgrowth of pathogenic bacteria (Lefere et al., 2020). Nitrate serves as an important respiratory substrate for enteric bacteria such as *Escherichia coli* and *Salmonella*. Activation of PPAR-γ also promotes β-oxidation, helping maintain the intestinal lumen in a hypoxic state and thereby limiting the growth of facultative anaerobes (Byndloss et al., 2017; Lee et al., 2022). Moreover, PPARγ signaling can suppress the pro-inflammatory M1 macrophage phenotype and promote metabolically adaptive macrophage polarization (Byndloss et al., 2017). In NAFLD models, activation of PPARγ alleviates lipid deposition and may indirectly reduce fibrosis by limiting hepatic stellate cell activation (Lefere et al., 2020).

Taken together, these interventions illustrate the therapeutic potential of targeting the bioenergetic–immunometabolic interface in SILI. These strategies can be broadly categorized into hepatocyte-directed metabolic rescue, immune-cell reprogramming, and system-level metabolic modulation, highlighting the multi-layered nature of immunometabolic targeting in SILI. However, most of these interventions remain supported primarily by preclinical evidence, with limited pharmacokinetic, safety, and efficacy data available in patients with sepsis-associated liver injury. Furthermore, it remains uncertain whether improvements in bioenergetic and immunometabolic parameters observed in experimental models can translate into clinically meaningful outcomes in patients with SILI. The therapeutic impact of immunometabolic modulation is also likely to depend on disease stage, because interventions that are beneficial during hyperinflammation may not exert similar effects during later immunosuppressive phases. Therefore, prospective studies are required to define the optimal timing, patient selection, and safety profiles of these metabolism-based interventions. Disease-stage-specific intervention windows and patient stratification therefore remain major barriers to clinical implementation. Emerging multi-omics approaches may help overcome. Recent advances in high-resolution multi-omics technologies, including single-nucleus RNA sequencing and spatial transcriptomics, have provided new opportunities to identify disease-specific cellular states and therapeutic targets. Mao et al. comprehensively reviewed the current state and future perspectives of single-cell omics for dissecting cellular heterogeneity in drug-induced liver injury (DILI), emphasizing its promise in elucidating disease mechanisms, improving risk prediction, and facilitating precision diagnosis (Mao et al., 2024). By integrating these approaches, Zhu et al. identified specialized subpopulations of astrocytes, microglia, and vascular cells in a mouse model of sepsis-associated encephalopathy, revealing previously unrecognized cellular interactions and immunometabolic regulatory networks (Zhu et al., 2024). Similar approaches may be applied to SILI to characterize hepatic cellular heterogeneity, identify disease-stage-specific targets, and facilitate the development of precision immunometabolic therapies.

### 6.3 Nanotechnology-enabled delivery systems

To overcome limitations in bioavailability and intracellular delivery, nanotechnology-enabled delivery systems have emerged as a promising adjunct to metabolism-based therapies. Rather than constituting a separate therapeutic concept, these platforms may enhance the feasibility and organ targeting of existing metabolic interventions. With regard to metabolic NAD^+^ deficiency, NAD^+^ is a critical coenzyme involved in both glycolysis and oxidative phosphorylation (Prolla and Denu, 2014). It is rapidly consumed during inflammatory stress (Qiu et al., 2023). Direct supplementation is often ineffective due to its negatively charged and hydrophilic nature, which hinders its diffusion across cell membranes. Moreover, cellular uptake is generally more efficient for NAD^+^ precursors such as nicotinamide (NAM) and nicotinamide riboside (NR) than for NAD^+^ itself (Qiu et al., 2023). In addition, the limited intracellular delivery efficiency of NAD^+^ necessitates high doses to maintain adequate intracellular levels, which restricts its clinical translation (Ye et al., 2022). However, newly developed NAD(H)-loaded nanoparticles (NPs) may overcome these limitations by enabling controlled release and protecting NAD^+^ from enzymatic degradation (Ye et al., 2022). This protective effect may be enhanced under acidic conditions through a proton-sponge mechanism (Ye et al., 2022). Additionally, NAD(H)-loaded nanoparticles exhibit favorable biocompatibility and can be effectively delivered to multiple cell types (Ye et al., 2022). NAD^+^-loaded lipid-coated calcium phosphate nanoparticles (NAD^+^-LP-CaP) exhibit high loading capacity and tend to accumulate in the liver because clearance efficiency is reduced during liver injury (Ye et al., 2022). In addition to limiting inflammatory progression by replenishing energy substrates, reducing inflammatory cytokine secretion, and suppressing pyroptosis and apoptosis, NAD^+^-LP-CaP can also be combined with the antibiotic rifampicin (Rif) to generate NAD^+^-Rif-LP-CaP (Y et al., 2021). This combination not only replenishes NAD^+^ but also delivers antibiotics to highly perfused target organs such as the liver and kidneys (Ye et al., 2022). In doing so, it may help control infection, maintain immune homeostasis, and reduce the risk of multiple organ failure (Ye et al., 2022). Nonetheless, issues related to large-scale manufacturing, *in vivo* targeting precision, pharmacokinetics, and long-term biosafety must be addressed before such systems can be widely translated. Moreover, evidence supporting nanotechnology-enabled delivery systems in sepsis and SILI remains largely preclinical, and no studies have established their efficacy or safety in critically ill patients with sepsis or SILI. Therefore, these platforms should currently be regarded as promising but experimental delivery strategies that may facilitate the translation of metabolism-based therapies rather than as established therapeutic modalities. Accordingly, scalable manufacturing, organ-specific targeting, and long-term biosafety remain the key bottlenecks preventing clinical translation.

## Conclusion

Mitochondrial dysfunction represents a key vulnerability point in sepsis-induced liver injury, integrating metabolic stress, immune dysregulation, and inflammatory amplification into a coordinated pathophysiological response. Rather than functioning as an isolated downstream consequence, it may reflect an early and dynamic shift in cellular adaptation failure during sepsis progression.

Despite advances in delineating these mechanisms, translation into clinical practice remains limited. Current biomarkers lack sufficient specificity and prospective validation in clinically stratified SILI populations, while most therapeutic strategies targeting metabolic or mitochondrial pathways have not yet demonstrated robust efficacy in human studies. These limitations highlight a persistent gap between mechanistic insight and clinically actionable interventions.

Bridging this gap will require a shift from pathway-centered descriptions to stage-aware and context-specific translational frameworks. Future efforts should prioritize clinically stratified validation cohorts, integration of temporal disease dynamics, and development of combination strategies that jointly address infection control, metabolic restoration, and immune modulation. Such approaches may enable more precise intervention windows and improve outcome prediction in SILI.
